# Repeated Cross-Sectional Survey of Ectoparasites in Sheep from Central Tunisia: Does Low Prevalence Indicate Good Hygiene or Resistance to Ectoparasites?

**DOI:** 10.3390/ani14050801

**Published:** 2024-03-04

**Authors:** Khawla Elati, Nesrine Daly, Mokhtar Dhibi, Hela Laaribi, Mourad Rekik, Mohamed Gharbi

**Affiliations:** 1 Laboratory of Parasitology, Institution of Agricultural Research and Higher Education, National School of Veterinary Medicine of Sidi Thabet, University of Manouba, Sidi Thabet 2020, Tunisia; dali.nessrine@gmail.com (N.D.); mokhtardhibi@gmail.com (M.D.); helalaaribi@gmail.com (H.L.); gharbim2000@yahoo.fr (M.G.); 2 Institute for Parasitology and Tropical Veterinary Medicine, Freie Universität Berlin, Robert-von-Ostertag-Str. 7, 14163 Berlin, Germany; 3 Veterinary Centre for Resistance Research, Freie Universität Berlin, Robert-Von-Ostertag-Str. 8, 14163 Berlin, Germany; 4 International Center for Agricultural Research in the Dry Areas (ICARDA), P.O. Box 950764, Amman 11195, Jordan; m.rekik@cgiar.org

**Keywords:** *Psoroptes ovis*, *Ctenocephalides canis*, ticks, ectoparasites, sheep

## Abstract

**Simple Summary:**

Sheep ectoparasites such as chewing lice, fleas and ticks are serious impediments to sheep productivity. They cause enormous losses of wool and leather due to the skin lesions they cause, which reduces the market value of sheep. Some of these ectoparasites are also vectors of several pathogens. In the present study, we aim to investigate the ectoparasite population infesting 1243 autochthonous sheep in Tunisia from two breeds, Barbarine and Queue Fine de l’Ouest. A total of 74 sheep (5.95%) were infested by 3 groups of ectoparasites. The low prevalence of ectoparasite infestation in sheep reported here may be due to possible genetic resistance or simply to successful hygiene and management measures implemented by farmers.

**Abstract:**

Sheep ectoparasites such as chewing lice, fleas and ticks are serious constraints to sheep productivity and are the cause of skin lesions in animals that decrease their market value. This study aims at investigating the ectoparasite fauna infesting small ruminants in the district of Sidi Bouzid (central Tunisia). A total of 1243 Barbarine and Queue Fine de l’Ouest (QFO) sheep were examined every two months for one year. Of the total animals examined, 74 were infested by at least 1 parasite group (5.95%). Three ectoparasite groups were identified as *Psoroptes ovis* (0.48%; 6/1243), ticks (5.3%; *n* = 66/1243) and one specimen of *Ctenocephalides canis* (0.08%; *n* = 1/1243). The most abundant tick among the 358 specimens was *Rhipicephalus sanguineus* sensu lato (*n* = 337; 94.1%), followed by *Hyalomma impeltatum* (*n* = 7/358; 1.9%), *H. dromedarii* (*n* = 7/358; 1.9%), *H. excavatum* (*n* = 5/358; 1.4%) and only two specimens of *H. scupense* (*n* = 2/358; 0.55%). The sheep herds showed low infestation prevalence by ectoparasite over the year, with a significant difference according to the seasons (*p* < 0.05). A higher infestation prevalence was recorded in March (14.36%). Barbarine sheep breed showed significantly higher infestation prevalence (16.8%) compared to QFO (0.8%) (*p* < 0.01). There were no differences in infestation prevalence according to sex of the animal or age groups. Knowledge of the ectoparasite population harboured by sheep, its activity dynamics and risk factors is required to develop effective ectoparasite control options. The low prevalence of ectoparasite infestation in sheep reported here may be due to possible genetic resistance or simply to successful hygiene measures implemented by farmers.

## 1. Introduction

As in several African countries, sheep husbandry in Tunisia is one of the most important agricultural activities. The sheep population is estimated to 6.485 million heads producing yearly approximately 123,000 tonnes of red meat [1]. This sector contributes significantly to the livelihood of farmers in rural areas, to employment and to a decrease in rural depopulation due to migration. In addition to dairy and red meat productions, sheep are also used for leather and wool production, hence providing 205,911 additional jobs in the textile sector [2]. However, these two industries are facing several constraints in terms of raw material availability and low quality due to several factors (malnutrition, parasitic infestations…) [3].

The Tunisian sheep population is mainly dominated by the indigenous fat-tailed Barbarine breed (64%), followed by thin-tailed breeds consisting of Queue Fine de l’Ouest (30%), Noire de Thibar (2%) and Sicilo-Sarde (0.5%) [4]. The Barbarine breed (locally called Nejdi or Arbi) was imported by the Phoenicians 400 BC during the Carthaginian period; it is adapted to different extreme climatic conditions and lack of forage due to the reservoir of energy in their tail, which weighs between 1.5 and 7 kg. This breed is found in all Tunisian bioclimatic zones [5], while the Queue Fine de l’Ouest is derived from the Ouled Jellal Algerian sheep breed; they are mainly found in the western part of the country, and they are adapted to cold temperatures and mountain grazing [6]. These two sheep breeds contribute to 23% of total meat production [6].

Sheep breeding activity is facing different challenges consisting of climate changes, which is leading to landscape cover modification and lack of forage in arid regions, which constitute most of the country surface. In addition to poor managing systems, sheep breeding is affected by the presence of a wide variety of parasites such as gastrointestinal helminths including *Trichostrongylus* spp., *Teladorsagia* spp., *Strongyloides papillosus* and Anoplocephalidea [7], lungworms [8] and haemoparasites such as *Babesia*, *Theileria* and *Anaplasma* [9].

Sheep ectoparasites, including ticks, lice, mange mites, sheep ked and ticks, are also causing major losses in quantity and quality of wool and leather [10]. Skin lesions (such as hyperkeratosis, acanthosis and follicular keratosis) are frequently complicated by bacterial infections, further hampering the economic and health impacts of these ectoparasites. The type of lesion depends on the species of ectoparasite and the reaction of the host to the infestation [11]. Parasitic dermatoses represent a large and important condition, although they are neglected by breeders, especially when only few animals are affected. These parasites can occur all over the year but are most active during the spring and the summer [12].

Hard ticks are blood-sucking ectoparasites that infest the widest range of hosts. These acari attach to thin skin, feeding on blood; a high infestation can lead to anaemia, skin irritation and infections. They are vectors of several pathogens (virus, bacteria and parasites) of medical, veterinary and economic importance, causing huge losses in livestock production [13]. In Tunisia, the tick fauna consists of 18 tick species belonging to 5 genera: *Hyalomma*, *Ixodes*, *Rhipicephalus*, *Haemaphysalis*, and *Dermacentor*. These ticks were collected from different bioclimatic zones and infested wild and domestic vertebrates [14,15]. Previous reports showed that sheep in Tunisia were mainly infested by ticks of the genera *Rhipicephalus* and *Hyalomma* [16,17].

In addition to ticks, other sheep ectoparasites may act as a vector for various pathogens. *Melophagus ovinus* (Diptera: Hippoboscoidea) or sheep ked are wingless flies. These arthropods are blood-sucking ectoparasites that transmit bacterial and protozoan pathogens such as *Rickettsia raoultii* and *R. slovaca* [18], *Borrelia burgdorferi* sensu lato, *Trypanosoma melophagium* and *Bartonella* spp. [19].

Certain mite parasites, such as the sarcoptic (*Sarcoptes scabiei*) and the psoroptic mites (*Psoroptes ovis*), cause ovine mange, also called sheep scab, which is highly contagious. It is characterized by loss of wool, scratching, bleeding wounds and loss of general condition [20]. These have no vector role but act as carriers for secondary bacterial infections due to the deep and chronic skin lesions they cause [20,21].

While some ectoparasites are not specific to sheep, they are transmitted to sheep when cohabiting with other domestic animals, such as carnivores. This is the case of fleas, *Ctenocephalides canis* and *Ctenocephalides felis* [22]. In Tunisia, these fleas are vectors of zoonotic agents such as *Bartonella* spp. [23].

The diversity of ectoparasites infesting sheep and their interactions with their hosts increase the constraints to control parasites. For instance, the control of ectoparasites (including ticks and mites acari) is often based on the use of acaricides such as formamidines (e.g., amitraz), phenylpyrazoles (e.g., fipronil), carbamates (e.g., carbaryl) and synthetic pyrethroids (e.g., flumethrin, deltamethrin, cypermethrin…), which have several disadvantages including toxicity to the host (residues in milk and meat) and to the environment (contamination of water and soil, impact on different soil invertebrates) [24]. In addition, several studies have shown that intensive use of acaricides leads to the emergence of resistance in different ectoparasite populations, which leads to the failure of control programmes [24]. The resistance appears when acaricides are used at subtoxic concentrations [25]. Multi-acaricide-resistant populations were reported in *R. appendiculatus* [26], *R. microplus* [27], *Hyalomma* ticks [28] and in the main sheep parasite arthropods including chewing lice, flies and ticks [29].

As an alternative to the use of chemicals to control ectoparasites, an integrated control programme including selecting sheep breeds that are not tick-attractive has been suggested as a sustainable tool, where some sheep breeds show lower tick burdens such as the indigenous fat-tailed Namaqua Afrikaner in South Africa [30] and Barbarine sheep in Tunisia [16]. Knowledge on host resistance to tick infestation is more rich in cattle [31,32,33,34]; this resistance is related to genetic and immunological parameters and is acquired after several exposures to tick infestations.

Some factors might contribute to the increase in ectoparasite infestation prevalence. For example, the illegal movement of animals across borders, and sharing pastures, which are known to be rich in certain food sources and which is also a common practice among most livestock farmers. This leads to infestations and infections spreading from carrier animals, sick animals and grazing areas where certain parasites can survive.

Climate change may also be another aggravating factor, as it is an important emerging risk factor affecting livestock health [35] and this is particularly relevant to North Africa, which is ranked as a hotspot for climate change [36]. Increases in temperature not only cause reductions in growth rate, milk yield and reproductive performance [37] but may affect the spread and the abundance of several vector arthropods such as ticks, mosquitos and flies and also other ectoparasites, leading to critical changes in transmission patterns of several vector-borne pathogens [38].

There is a lack of studies on the diversity of sheep ectoparasites in Tunisia, their biology and their economic impact on sheep productivity, except studies that were focusing on ticks [16,17,39]. Managing ectoparasites in Tunisian sheep flocks requires a combination of preventive measures, including regular monitoring and knowledge of regional parasite prevalence, which are crucial for effective management strategies. Therefore, the aim of the present study was to estimate the prevalence of sheep ectoparasites and their associated risk factors in a major sheep production region in central Tunisia.

## 2. Materials and Methods

### 2.1. Study Area

**Figure 1:**
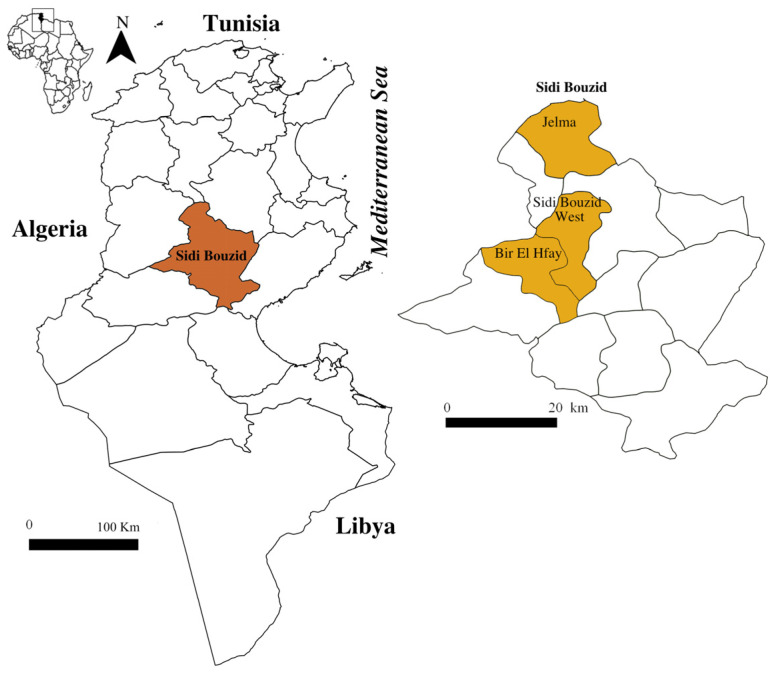
Geographic localisation of Sidi Bouzid district in Tunisia (Author’s source, Qgis software version 3.8.2).

The present study was carried out in the district of Sidi Bouzid (central Tunisia), which has an average altitude of 327 m (Figure 1).

This region is characterised by a semi-arid to arid climate with an average annual rainfall of 234 mm and monthly maximum and minimum temperatures of 40 and 2 °C in July and January, respectively (Climate-Data.org Accessed 17 March 2023). Agriculture is the main economic activity in the district; Sidi Bouzid is the first district for vegetables’ production in Tunisia and has an important livestock activity with a sheep population estimated to 662,200 heads [40].

### 2.2. Animals and Samples

A total of 1243 animals belonging to six randomly selected sheep flocks from three localities in the Sidi Bouzid district (Jelma, Bir El Hfay and Sidi Bouzid West) were surveyed every two months for one year from September to July. The distance between the farms visited is less than 41 km. Four flocks were reared in an extensive system, one in a semi-intensive system. In the last flock, farmers used an intensive system for animals that were imported from Algeria every two months and an extensive system for indigenous sheep. The sheep cohabited with cattle, goats, dogs and poultry. The treatment of animals with acaricides varied from one farm to another (Table 1).

The size of the herds varied between 144 and 268 heads. The monitored animals consisted of 736 females and 507 males, of which 363 were of the Barbarine breed and 880 of the Queue Fine de l’Ouest breed. The sheep were divided into six age groups (less than 1 year, between 1 and 2 years, between 2 and 3 years, between 3 and 4 years, between 4 and 5 years, between 5 and 10 years). The majority of animals were less than 1 year old (*n* = 871).

All animals were thoroughly examined for ectoparasites by a veterinarian with the help of the sheep owners. Parasites were collected and preserved in labelled vials containing 70% ethanol. Ticks were identified under a binocular stereoscope using different keys [14,41]. Other ectoparasites were identified as described by Wall et al. [42].

### 2.3. Parasitological Parameters and Statistical Analysis

The infestation prevalence was estimated as follows [43]:

Infestation prevalence (%) = 100 × (number of infested animals/number of examined animals)

Comparisons of infestation prevalence between farms, age groups, sexes and breeds were made using the chi-squared test at 5% threshold using SPSS software (v. 21, IBM, Armonk, NY, USA) [44].

## 3. Results

A total of three groups of ectoparasites were collected: ticks, *Psoroptes ovis* and *Ctenocephalides canis*. No sheep ked (*Melophagus ovinus*) were collected from any of the animals examined.

The overall infestation prevalence with at least one of these parasites was 5.95% (74/1243). *Psoroptes ovis* was the only scabies parasite identified; it was collected from six animals belonging to three farms (6/1243; 0.48%). Only one flea specimen, identified as *Ctenocephalides canis*, was collected from a Que Fine de l’Ouest (QFO) male from Farm 2 in September (1/1243; 0.08%). A total of 358 adults ticks were collected, belonging to 5 tick species, of which *Rhipicephalus sanguineus* sensu lato (*n* = 337/358; 94.1%) was the most abundant, followed by *Hyalomma impeltatum* (*n* = 7/358; 1.9%), *Hyalomma dromedarii* (*n* = 7/358; 1.9%), *Hyalomma excavatum* (*n* = 5/351; 1.4%) and *Hyalomma scupense* (*n* = 2/358; 0.55%) (*p* < 0.01). The tick infestation prevalence in Barbarine sheep was significantly higher than in QFO breeds (16.8 and 0.57%, respectively, *p* < 0.01). Farm 3 was the most infested by ticks compared to the other farms, where 47.2% (60/127) of the sheep were infested (*p* < 0.0001) (Table 2).

**Figure 2:**
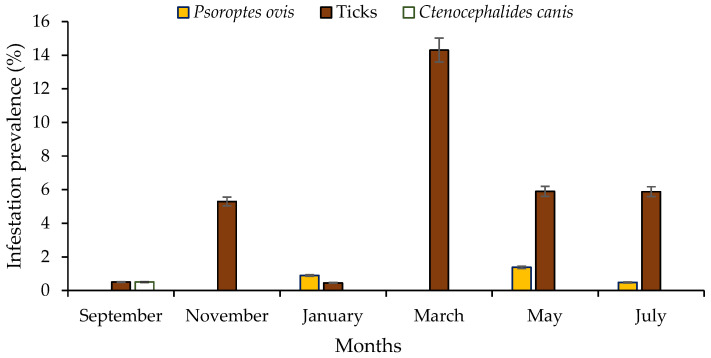
Monthly infestation prevalence of sheep by ectoparasite species in Sidi Bouzid region (Central Tunisia). Bars: standard error.

Sheep were more infested by ticks than other ectoparasites during all the visits, with a higher infestation prevalence in March (66/1243; 14.3%) (*p* < 0.0001) (Figure 2).

There was a statistically significant difference between the prevalence of ectoparasite infestation according to season, breed and farm (*p* < 0.0001), but there was no statistical difference according to sex (*p* = 0.4) and age group (*p* = 0.08) (Table 2).

**Figure 3:**
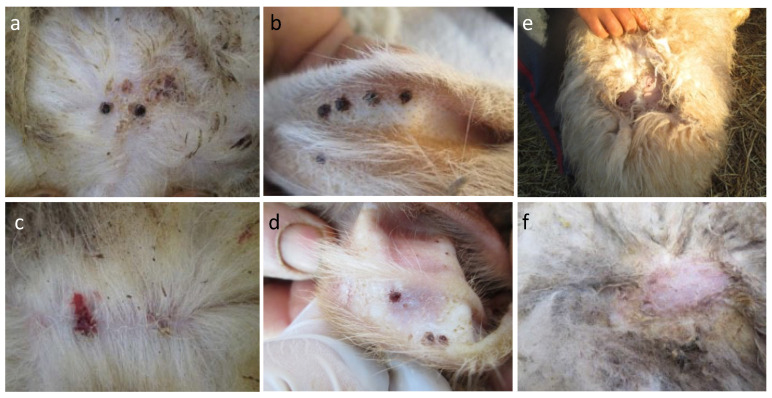
Tick attachment sites (**a**,**b**) and skin lesions due to ticks (**c**,**d**) and *Psoroptes ovis* (**e**,**f**).

Ticks were collected from the ears and from the sternum of animals (Figure 3a,b). Skin lesions due to ticks (Figure 3c,d) and *P. ovis* (Figure 3e,f) were observed on infested sheep.

## 4. Discussion

Several ectoparasites affect small ruminants and cause serious skin diseases that decrease farm financial income due to market value decrease of sheep with skin lesions. Some of the ectoparasites such as ticks represent a risk to human health (anaplasmosis and Mediterranean spotted fever) [45,46] and/or limit animal trade (theilerioses, babesioses…) [47,48].

The present study aimed at estimating the prevalence of ectoparasites infestation in sheep from Sidi Bouzid district strategically located in the Tunisian sheep production belt. Out of 1243 examined animals, only 74 (5.95%) were infested by at least one parasite. This result is consistent with those reported from South Benin [49] and Iran [50] with overall infestation prevalence of 7.8 and 8.2%, respectively. Infestation prevalence estimated herein is much lower than those reported in Northwest Ethiopia (47.7% [51] and 48.9% [52]) and Iraq (57.7%) [53]. The difference may be due to the geographic location, farming system, management and husbandry practices, malnutrition, host susceptibility and the prevailing climate, which may affect the development of these parasites. In addition, the difference of knowledge about the impact of these parasites by sheep owners may dramatically influence the animals’ infestation status [54,55]. The herd size and density can be a factor influencing the infestation prevalence since the overcrowding of the herd can facilitate the spread of ectoparasites, increasing their prevalence within a flock.

Three parasite groups were identified in this study; ticks (5.3%) were the most abundant, followed by *Psoroptes ovis* (0.48%), and only one specimen of *C. canis* was collected. The ectoparasite fauna and abundance varies between the studies. This trend in tick abundance compared to other ectoparasites is similar to those reported from Ethiopia, Iran and Iraq, where ticks were the main collected ectoparasites (31.8, 90 and 46.7%, respectively) [50,53,56]. The ectoparasite community assemblage is influenced by biotic and abiotic factors, which means that there are host-related factors (host species, gender, age, breed and immune system) and environment-related factors (temperature, humidity and human disturbance) [57]. In addition, several studies focused on interactions between different ectoparasites co-infesting an animal [58,59]. The dominance of ticks found in this study could be explained by an antagonistic competitive interaction mediated by physical or chemical signals, as it has been reported previously for several parasites such as chiggers, ticks, fleas and lice [58]. The diversity of the group of ectoparasites that infests sheep means a diversity in their life cycles, which therefore affects the effectiveness of the implemented control measures. In the case of parasites that are permanently present on the surface of the host (sarcoptic and psoroptic mites and chewing lice), veterinary drugs can control almost the entire population from the first infestation. In the case of parasites that are only temporarily present on the host, such as three-host ticks, the population living on the host is rapidly replaced by individuals living in the environment. In addition, the sensitivity of ticks to control measures decrease with the number of hosts (two or three-host tick species) [60].

Barbarine animals were significantly more infested by ectoparasites (16.8%) than QFO animals (0.8%) (*p* < 0.0001). This result is in contrast with those reported by Rjeibi et al. [61], who did not find any difference in infestation prevalence by ticks between Barbarine and Queue Fine de l’Ouest breeds in Northwest Tunisia (humid region). Indeed, in Siliana district (Northwest of Tunisia), Barbarine sheep were significantly less infested by ticks (7.3%) compared to cross-bred (19.1%) and QFO (16.7%) sheep. A possible explanation for this difference could be the lower attractiveness or a higher resistance to ticks of some sheep breeds or ecotypes [16] or also to difference in sampling protocol between the studies.

Significant variation in ectoparasite infestation was reported between farms (*p* < 0.0001). This may be related to differences in implementing control measures (Table 1), sheep flock size and density, husbandry system and cohabitation with other animals.

The way animals are reared is an important factor in their susceptibility to ectoparasites. In fact, the problem of self-medication is becoming widespread in addition to the development of parallel markets selling veterinary medicines at lower prices, but low quality might lead to an increase in the resistance in animals to molecules designed to control ectoparasites. The presence of cracks and crevices in the walls and the persistence of organic matter in and around the sheepfold favour the development and survival of some parasites [62].

Animals from Farm 3 were the most infested by ectoparasites (50.3%), with dominance of ticks (47.2%) belonging to five species dominated by *R. sanguineus* sensu lato (*n* = 337/358; 94.1%), followed by *H. impeltatum* (*n* = 7/358; 1.9%), *H. dromedarii* (*n* = 7/358; 1.9%), *H. excavatum* (*n* = 5/351; 1.4%) and *H. scupense* (*n* = 2/358; 0.55%). This farm was located in a highland containing grass shared by several sheep flocks, enhancing the risk of parasites’ transmission. It is worth mentioning that sheep in this farm were living with dogs, which may explain the dominance of *R. sanguineus* s.l. ticks (94.1%). This finding confirms previous studies in Tunisia investigating the infestation of sheep by ticks [16,17,39]. In Constantine, Northeast Algeria, *Rhipicephalus* was also the main genus infesting sheep, where *R. bursa* was the most prevalent tick species (88.6%) [63]. This result confirms those reported by Ramezani et al. [64] in mountainous areas in Iran, where tick infestation prevalence was estimated to 59.7%. The presence of *H. dromedarii* could be explained by a cohabitation with dromedaries, as they are the preferential host of this tick species [65]. The same authors mentioned that there is a high risk that *H. dromedarii* becomes adapted to sheep since the two mammal species share the same pastures. The dominance of *Rhipicephalus* ticks recorded in this study is not surprising, as it has been reported that this genus is the most prevalent infesting domestic small ruminants in Africa with twenty-seven species [66].

*Rhipicephalus* and *Hyalomma* ticks were collected from the ears and the sternum, respectively, as previously reported [17]. Knowledge of tick attachment sites helps farmers in reducing acaricide use and an easier and more efficient manual tick removal.

Ectoparasites are the main cause of various skin lesions in animals. Two types of skin lesions and defects were observed during our study. The first type was caused by tick infestation (Figure 3c,d); it was characterized by bleeding spots and crusts around the attachment sites where ticks secrete cement to anchor their mouthpart to the host skin during the blood meal. The second type was gross lesions caused by *Psoroptes ovis* resulting in wool loss (Figure 3e,f), which is extremely contagious, and the growth and the size of the lesions are related to the sheep breed and the infestation duration [67]. These two types of lesions found here have been reported by Chanie et al. in Ethiopia [11].

The present study showed no significant difference in infestation prevalence according to the sex (*p* = 0.4) and age group (*p* = 0.08) of the sheep. This could be explained by fluctuations in the sample structure between studies. Indeed, previous studies showed higher infestation prevalence in female (75.4%) compared to male sheep (61.5%), which could be attributed to the physiological status of the female, as both pregnancy and lactation cause an immunodepression and therefore a higher susceptibility to tick infestation [56].

Tick infestation showed a statistically significant seasonal pattern. In fact, 14.3% of the ticks were collected in spring (March) and 5.9% in summer (July) (*p* < 0.0001). The difference in activity dynamics could be explained by the abiotic factors. Indeed, Tunisia has a Mediterranean climate (Climate-Data.org, Accessed 17 March 2023). There are four seasons, with winter being the coldest and wettest and summer the hottest and driest. Previous studies monitoring the activity dynamics of *H. scupense* on cattle and *H. dromedarii* on dromedaries under field conditions showed that their activities are seasonal [15]. *R. sanguineus* was collected from sheep between April and September, with few specimen in October [17], which confirms its higher abundance during warmer months compared to winter [68]. The seasonality of ticks’ activity and their geographical distribution are determined by environmental factors [69,70]. Knowledge of this aspect is useful for the implementation of effective control programmes since developmental stage, seasons, host species and attachment site are often closely correlated.

## 5. Conclusions

Current work showed a low infestation prevalence of autochthonous sheep breeds in Tunisia by different ectoparasites. The significant variation of infestation among farms shows on the one hand the successful implementation of current control measures by farmers. In addition, the low infestation reported herein suggests that both sheep breeds may have less susceptibility to ectoparasite infestation and may be specifically considered as tick-resistant and could be included in genetic selection programmes. A good knowledge of the ectoparasite populations and their biology is of paramount importance for the implementation of any effective control programme against these parasites. Good livestock management should be recommended in order to reduce the losses due to ectoparasites infestation and their control.

## Figures and Tables

**Table 1:** Characteristics of the farms monitored and prophylactic management.

Farm	Production System	Sheep Breed	Sympatric Animals	Antiparasitic Treatments	Ectoparasites Found
1	Semi-intensive	Barbarine	Goat and poultry	- Ivermectin every six months - Spraying acaricide after shearing the animals	*Psoroptes ovis* Ticks
2	Intensive for fattening flocks and extensive for the original flock	QFO	Goat, cattle, poultry and dogs	- Monthly treatment, with skin parasitosis cases appearing after each treatment (using ivermectin imported from Algeria). - Ivermectin treatment of newly introduced animals - Acaricide by bathing after animals’ shearing	*Psoroptes ovis* *Ctenocephalides canis*
3	Extensive	Barbarine	Dogs	- Antiparasitic treatment every 6 months - Treatment every time a skin parasitosis case appears - Acaricide by bathing after animals’ shearing	Ticks *Psoroptes ovis*
4	Extensive	QFO	Poultry	- Ivermectin and diazinon - Treatment of the barn with acaricide	Ticks
5	Extensive	Barbarine and QFO	Goats	- Treatment of animals each time a skin parasitosis case appears (ivermectin imported from Jordan) - Good hygiene of barns and animals	Ticks
6	Extensive	QFO	Goats	- Treatment with ivermectin whenever a skin parasitosis case appears - Good hygiene of barns and animals	0

**Table 2:** Ectoparasite infestation prevalence according to different risk factors.

	Infested/Examined (Infestation Prevalence in % ± Standard Error)			
Parameters	*Psoroptes ovis*	Ticks	*Ctenocephalides canis*	Overall
Farm				
1	1/236 (0.4 ± 0.8)	1/236 (0.4 ± 0.8) *	0/236 (0)	2/236 (0.9 ± 1.2) *
2	1/260 (0.3 ± 0.8)	0/260 (0)	1/260 (0.4 ± 0.8)	3/260 (1.1 ± 1.3)
3	4/127 (3.2 ± 3)	60/127 (47.2 ± 8.7)	0/127 (0)	64/127 (50.3 ± 8.7)
4	0/208 (0)	4/208 (1.9 ± 1.9)	0/208 (0)	4/208 (1.9 ± 1.9)
5	0/144 (0)	1/144 (0.7 ± 1.4)	0/144 (0)	1/144 (0.7 ± 1.4)
6	0/268 (0)	0/268 (0)	0/268 (0)	0/268 (0)
Gender				
Female	5/736 (5 ± 0.7)	42/736 (5.7 ± 1.7)	0/736 (0)	47/736 (6.4 ± 1.8)
Male	1/507 (1 ± 0.2)	24/507 (4.7 ± 1.9)	1/507 (0.2 ± 0.4)	27/507 (5.3 ± 2)
Breed				
Barbarine	5/363 (5 ± 1.4)	61/363 (16.8 ± 3.9) *	0/363(0)	67/363 (18.4 ± 4) *
Queue Fine de l’Ouest (QFO)	1/880 (1 ± 0.1)	5/880 (0.6 ± 0.5)	1/880 (0.1 ± 0.2)	7/880 (0.8 ± 0.6)
Age (years)				
[0–1]	2/871 (0.2 ± 0.3)	37/871 (4.3 ± 1.3)	1/871 (0.1 ± 0.2)	41/871 (4.7 ± 1.4)
[1–2]	2/108 (1.9 ± 2.5)	6/108 (5.6 ± 4.3)	0/108 (0)	8/108 (7.4 ± 2.5)
[2–3]	2/130 (1.5 ± 2.1)	10/130 (7.7 ± 4.6)	0/130 (0)	12/130 (9.2 ± 2.5)
[3–4]	0/109 (0)	11/109 (10.1 ± 5.7)	0/109 (0)	11/109 (10.1 ± 2.9)
[4–5]	0/20 (0)	2/20 (10 ± 13.2)	0/20 (0)	2/20 (10 ± 13.2)
[5–10]	0/5 (0)	0/5 (0)	0/5 (0)	0/5 (0)
Overall	6/1243 (0.48 ± 0.2)	66/1243 (5.3 ± 0.6)	1/1243 (0.08 ± 0.2)	74/1243 (5.95 ± 0.7)

* Statistically significant difference.

## Data Availability

All data generated or analysed during this study are included in this published article.
